# The Abundance of Toxic Genotypes Is a Key Contributor to Anatoxin Variability in *Phormidium*-Dominated Benthic Mats

**DOI:** 10.3390/md15100307

**Published:** 2017-10-11

**Authors:** Susanna A. Wood, Jonathan Puddick

**Affiliations:** 1 Cawthron Institute, Nelson 7010, New Zealand; susie.wood@cawthron.org.nz; 2 Environmental Research Institute, University of Waikato, Hamilton 3216, New Zealand

**Keywords:** benthic cyanobacteria, cobble-bed rivers, cyanotoxins, digital droplet PCR, liquid chromatography-mass spectrometry

## Abstract

The prevalence of benthic proliferations of the anatoxin-producing cyanobacterium *Phormidium* are increasing in cobble-bed rivers worldwide. Studies to date have shown high spatial and temporal variability in anatoxin concentrations among mats. In this study we determined anatoxin quotas (toxins per cell) in field samples and compared these results to the conventionally-used concentrations (assessed per dry weight of mat). Three mats were selected at sites in two rivers and were sampled every 2–3 h for 24–26 h. The samples were lyophilized and ground to a fine homogenous powder. Two aliquots of known weights were analyzed for anatoxin congeners using liquid chromatography-mass spectrometry, or digital droplet PCR with *Phormidium*-specific *anaC* primers to measure absolute quantities of gene copies. Anatoxin concentrations in the mats varied 59- and 303-fold in the two rivers over the study periods. A similar pattern was observed among gene copies (53- and 2828-fold). When converted to anatoxin quotas there was markedly less variability (42- and 16-fold), but significantly higher anatoxin quotas were observed in mats from the second river (*p* < 0.001, Student’s *t*-test). There were no obvious temporal patterns with high and low anatoxin concentrations or quotas measured at each sampling time and across the study period. These results demonstrate that variability in anatoxin concentrations among mats is primarily due to the abundance of toxic genotypes. No consistent modulation in anatoxin production was observed during the study, although significant differences in anatoxin quotas among rivers suggest that site-specific physiochemical or biological factors may influence anatoxin production.

## 1. Introduction

Proliferations of toxin-producing benthic cyanobacteria are being reported with increasing frequency in lakes, ponds, and rivers worldwide [1,2,3,4,5,6]. Most studies have focused on identifying the causative species and toxin, and knowledge on spatial and temporal variability in toxin concentrations is limited [1]. This is in stark contrast to planktonic cyanobacterial blooms, where these variables have been investigated for many decades (e.g., [7,8,9,10]). 

Of particular concern, among benthic species, is an increase in proliferations of mat-forming *Phormidium* in cobble-bed rivers worldwide [11,12,13,14]. This type of cyanobacteria commonly produces neuromuscular-blocking anatoxin-a (ATX) and homoanatoxin-a (HTX), and their structural variants dihydro-anatoxin-a (dhATX) and dihydro-homoanatoxin-a (dhHTX; hereafter collectively referred to as anatoxins) [1]. Although less commonly detected than other cyanotoxins (e.g., microcystins, cylindrospermopsin, saxitoxin) animal deaths linked to ingestion of mats containing anatoxin-producing *Phormidium* have been reported globally (e.g., France [11]; New Zealand [15]; United States of America [16], and The Netherlands [17]).

In New Zealand, studies have observed significant temporal shifts in anatoxin concentrations of *Phormidium autumnale*-dominated mats [18] (hereafter referred to as *Phormidium*) when pooled samples from 5–10 mats collected from the same site were assessed on a weekly basis (Heath et al. [19], ca. 27-fold; Wood et al. [20], ca. 80-fold; McAllister et al. [21], ca. 30-fold). Wood et al. [22] analyzed 15 mat samples from 10 × 10 grids in seven rivers and showed there was also marked spatial variability between and within sites. Samples from two sites contained no anatoxins, and at one site anatoxins were detected in all samples. At four sites both toxic and non-toxic samples co-occurred and these samples were sometimes spaced less than 1 m apart.

Three plausible theories have been put forward to explain the variability of anatoxin concentrations observed in *Phormidium* mats: (i) The relative abundance of co-occurring organisms and/or inorganic material vary in the mats. Although dominated by *Phormidium*, these mats also contain many other organisms (e.g., bacteria and eukaryotic algae), and inorganic matter, (e.g., sediment), bound together by extracellular polymeric substances [23,24,25]. The quantity and composition of these additional components can vary over time and between sites [19]; (ii) Conditions within the mat (e.g., dissolved oxygen, pH) or the surrounding water (e.g., nutrient concentrations) cause an up- or down-regulation in the amount of anatoxins produced per cell. Using culture-based studies Heath et al. [26] showed increased nitrogen and phosphorus concentrations resulted in lower ATX and HTX quotas (the amount of toxin produced per cell). However, this scenario is unlikely to explain the previously observed differences in anatoxin levels within close proximity as mats would be exposed to relatively similar environmental conditions; (iii) Toxic and non-toxic genotypes co-occur in the mats, and their relative abundance and amount of toxin they produce affect the total anatoxin concentrations. Evidence from Wood et al. [27] supports this suggestion. The authors isolated and cultured multiple *Phormidium* strains from 1 cm^2^ areas taken from four different mats collected in two rivers. Molecular and chemical analysis of the cultures showed that both toxic and non-toxic genotypes co-existed, and that among toxic strains the concentration of toxin produced per unit biomass varied by approximately 100-fold. Variables that regulate the presence and abundance of each genotype within *Phormidium* mats are unknown [27]. Several, or all, of these scenarios may be contributing to the overall anatoxin concentrations within a mat sample and to spatial and temporal shifts in toxin concentrations observed at a single site. 

Whilst culture-based studies have provided valuable insights into toxin-production in benthic mats, there is an increasing awareness that cyanobacteria need to be studied in the environment to improve knowledge on natural variability and toxin production [10,28]. Unlike water column samples (i.e., planktonic cyanobacteria bloom samples), it is not possible to normalize toxin concentrations to a known volume when working on benthic mats. *Phormidium*-dominated mats can vary in the amount of water they contain, depending on the mat composition and desiccation status when sampled. To overcome this problem, samples are typically lyophilized and the data is normalized to the dried weight; thus, other co-occurring organisms and inorganic material are included in these values. Although microscopic identification and enumeration of *Phormidium* filaments is possible, often the entangled filaments within the mats make obtaining a homogeneous sample difficult, and differentiating actual cells within the filaments is impossible without high magnification (1000×). Additionally, as occurs with other cyanobacteria, toxic and non-toxic genotypes cannot be distinguished using microscopic techniques [29].

The aims of the present study were: (1) to develop a method to determine anatoxin quotas in field samples; (2) to assess how anatoxin quotas varied over diurnal cycles; and (3) to determine which of the above three theories contributed to the observed differences in anatoxin concentrations and quotas. Three mats were selected at a single site in two rivers, and these were sampled repeatedly every 2–3 h for 24–26 h. We chose to sample over a diurnal cycle as previous studies have shown that the *Phormidium* mats experience extreme fluctuations in physicochemical parameters (e.g., pH and dissolved oxygen) over light and dark periods [25], and research on planktonic cyanobacteria has shown associations between toxin production and shifts in photosynthesis, reactive oxygen species, and pH [9,10,30].

## 2. Results

A method was developed to enable anatoxin quotas to be determined in *Phormidium* mat samples. Lyophilized mat samples were ground into a homogenous fine powder and two aliquots of each sample were weighed. Anatoxins were extracted from one aliquot and their concentration determined by liquid chromatography-tandem mass spectrometry (LC-MS/MS). Deoxyribonucelic acid (DNA) was extracted from the second aliquot and the concentration of *Phormidium autumnale anaC* gene copies were assessed using digital droplet PCR (ddPCR). Due to good sample homogeneity (from using a finely-ground lyophilized powder), and because both measurements were normalized to the weight of sample extracted, an anatoxin quota could be calculated.

**Figure 1:**
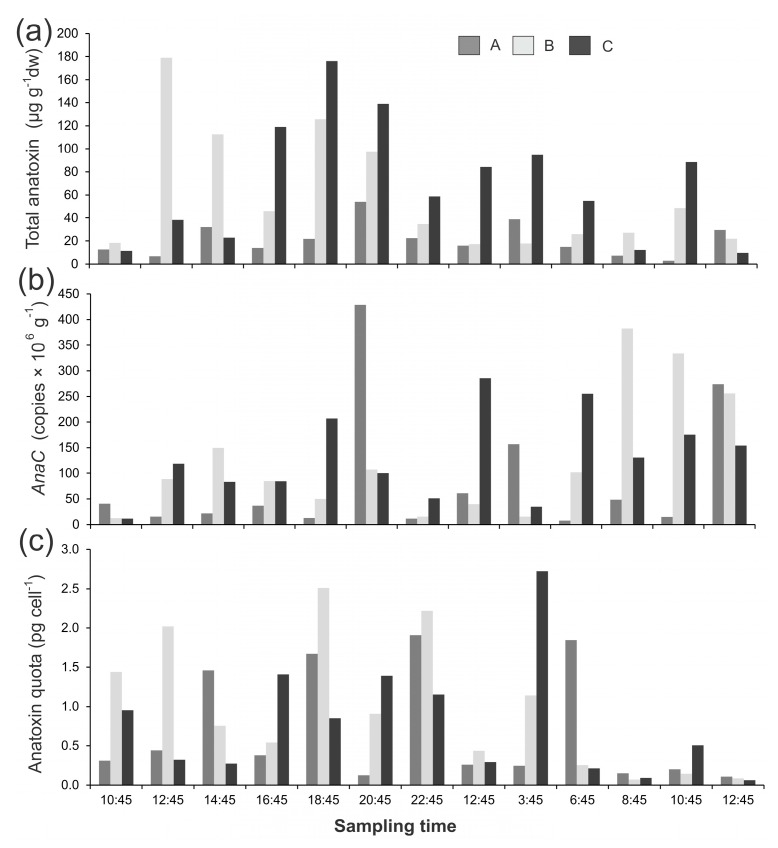
(**a**) Summed anatoxin (i.e., the total of the four congeners; anatoxin-a, homoanatoxin-a, dihydro-anatoxin-a, and dihydro-homoanatoxin-a); (**b**) concentrations of *anaC* copy numbers; and (**c**) anatoxin quotas from three *Phormidium* mat samples (A,B,C) collected from the Cardrona River on 10 and 11 April 2017. dw = dried weight. Note the different *y*-axis scales.

*Phormidium* mat samples were collected over a diurnal period from the Cardrona River and the Mataura River (New Zealand). The anatoxin concentrations in the Cardrona River mat samples showed large variations (up to 59-fold) when assessed per dry weight (dw; 3.0–179 µg g^−1^ dw; Figure 1a). At some time points (e.g., 10:45, 10 April 2017) the three different mats sampled had similar anatoxin concentrations, whereas at other sampling times there was large variability in anatoxin concentrations (e.g., 12:45, 10 April 2017; Figure 1a). There were no obvious temporal patterns with high and low anatoxin concentrations measured across the study period (Figure 1a). A very similar pattern was observed among gene copies with a 53-fold difference across the study period, and the within and among mat variability was similarly large (Figure 1b). When the anatoxin concentrations were converted to anatoxin quotas (i.e., the amount of anatoxin per toxic cell) there was less variability among samples (42-fold), but there were also no obvious temporal patterns (Figure 1c). There was a weak, but significant, relationship between gene copy numbers and anatoxin quotas (*R*^2^ = 0.27, *p* < 0.001).

**Figure 2:**
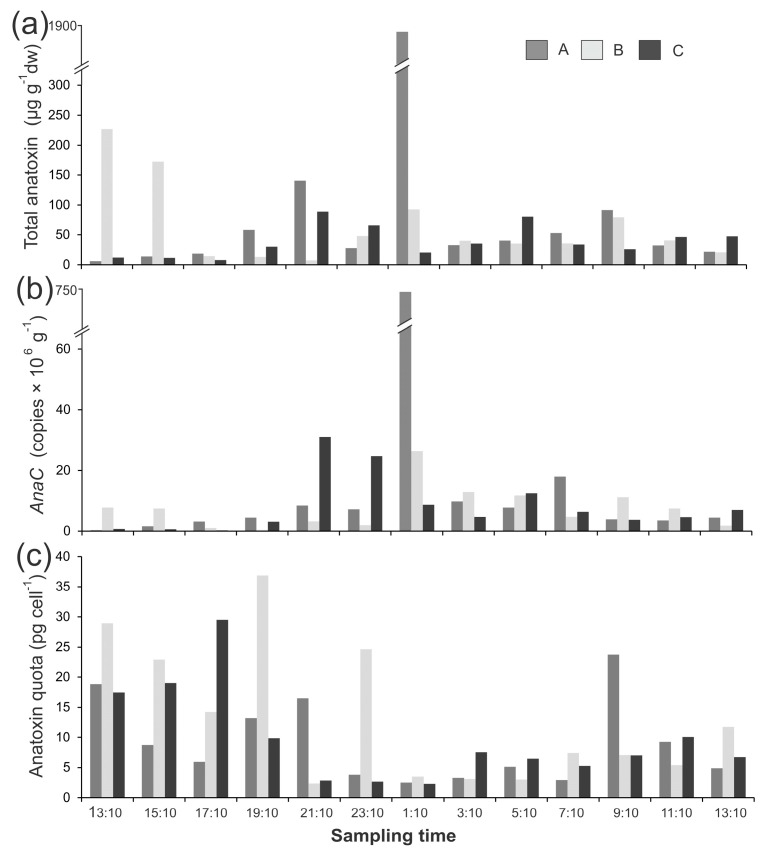
(**a**) Summed anatoxin (i.e., the total of the four congeners; anatoxin-a, homoanatoxin-a, dihydro-anatoxin-a, and dihydro-homoanatoxin-a); (**b**) concentrations of *anaC* copy numbers; and (**c**) anatoxin quotas from three *Phormidium* mat samples (A,B,C) collected from the from the Mataura River on 12 and 13 April 2017. dw = dried weight. Note the different *y*-axis scales, and the break in *y*-axes of A and B.

Anatoxin concentrations varied 303-fold in the mat samples collected from the Mataura River, which was largely due to one sample (Mat B, 01:10, 13 April 2017, 1889 µg g^−1^ dw; Figure 2a). There were no notable patterns among mats; i.e., toxin concentrations were not consistently higher in one mat and there were no obvious temporal shifts (Figure 2a). There was a very large difference among gene copies in the Mataura mats (2828-fold), which was (again) primarily due to Mat B 01:10 (750 × 10^6^ *anaC* copies g^−1^; Figure 2b). When converted to anatoxin quotas there was markedly less variability among samples (16-fold; Figure 2c). The most remarkable change, upon conversion to anatoxin quotas, was for Mat B 01:10 sample, where the anatoxin quota (2.5 pg cell^−1^) was on-par with concentrations in other mats (Figure 2c). There was a weak but significant relationship between gene copy numbers and anatoxin quotas (*R*^2^ = 0.24, *p* < 0.001).

The mean toxin concentration in the Cardrona mats (50.1 µg g^−1^ dw) was lower than the Mataura mats (96.3 µg g^−1^ dw), but this difference was not significant (*p* > 0.05, Student’s *t*-test). The mean anatoxin quota was significantly lower in the Cardrona mats (0.44 pg cell^−1^) compared to the Mataura mats (7.5 pg cell^−1^; *p* < 0.001, Student’s *t*-test).

**Figure 3:**
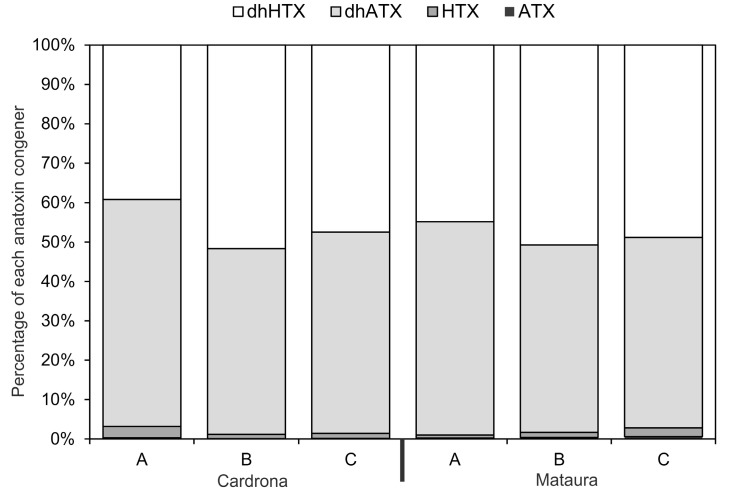
The percentage of each anatoxin congener in the three mats samples from each study site (data are an average of the 13 samples collected from each mat; ATX = anatoxin-a, HTX = homoanatoxin-a, dhATX = dihydro-anatoxin-a, dhHTX = dihydro-homoanatoxin-a).

The most abundant anatoxin congeners in the mat samples collected from the Cardrona and Mataura rivers were dhATX and dhHTX (Figure 3). Anatoxin-a and HTX were only present at low concentrations (Figure 3). The anatoxin congener composition of the three mats from the Mataura River was very similar, whilst one of the mats from the Cardrona River had a slightly different composition from the other two (i.e., a higher proportion of dhATX than the other two).

Over the sampling period water temperature in the Cardrona River varied between 12.0–13.9 °C, and in the Mataura River it varied between 11.1–12.6 °C (Supplementary Information Figure S1). Dissolved oxygen levels (means of 74% at Cardrona, and 103% at Mataura), conductivity (means of 108.6 µS cm^−1^ at Cardrona, and 119.8 µS cm^−1^ at Mataura) and pH (means of 7.2 at Cardrona, and 7.5 at Mataura) remained relatively constant across the sampling periods (Supplementary Information Table S1). The mean dissolved inorganic nitrogen (DIN) concentrations (sum of NO_2_-N, NO_3_-N, and NH_4_-N; *n* = 13) in the Cardrona and Mataura rivers were 0.324 mg L^−1^ (range of 0.136–0.464 mg L^−1^) and 0.577 mg L^−1^ (range 0.100–1.114 mg L^−1^), respectively. All dissolved reactive phosphorous (DRP) measurements in the Cardrona River, except one (0.004 mg L^−1^), were below the analytical limit of detection. The mean DRP concentration in the Mataura River was 0.007 mg L^−1^ (range 0.005–0.011 mg L^−1^; Supplementary Information Table S1). Student’s *t*-tests showed a significant difference in DIN (*p* = 0.002) concentrations between the two rivers. The mean water velocity was 0.07 m^3^ s^−1^ in the Cardrona River and 0.1 m^3^ s^−1^ in the Mataura River.

Linear regression suggested there was no relationship between the Cardrona River anatoxin quotas or gene copy numbers, and the measured physiochemical variables (all *R*^2^ < 0.09, *p* > 0.05). There was a very weak, but significant, relationship between anatoxin quotas or gene copy numbers, and conductivity in the Mataura River samples (*R*^2^ < 0.17, *p* < 0.01; *R*^2^ < 0.13, *p* < 0.05; respectively), all other relationships were non-significant (all *R*^2^ < 0.07, *p* > 0.05).

## 3. Discussion

The first aim of this study was to develop a method that enabled anatoxin quotas to be determined in *Phormidium*-dominated mat samples. The developed method involved lyophilizing mat samples and grinding them into a homogenous fine powder. Two aliquots of each sample were weighed out and extracted for DNA and for anatoxins. The concentration of anatoxin congeners was determined by LC-MS/MS and the concentration of *Phormidium autumnale anaC* gene copies in the extracted DNA was assessed using digital droplet PCR (ddPCR). Digital droplet PCR is a type of quantitative PCR that provides increased sensitivity, superior quantification, and also negates the need for standard curves and inhibition controls [31,32]. As both measurements were normalized to the weight of sample, an anatoxin quota could be determined. Although similar methods have been used to assess toxin quotas in planktonic cyanobacteria [28,33], this is the first study to apply the methodology to investigate anatoxin quotas in benthic cyanobacterial mats. The method proved to be relatively straightforward to implement, although caution must be applied when working with the fine lyophilized powder that can be easily aerosolized (e.g., the use of respirator masks and controlled working spaces is recommended).

The anatoxin concentration data conformed to patterns observed in other studies, with considerable spatial variability between mats and among sites, and the occasional sample with extremely high or low concentrations [20,21,22]. All samples were dominated by the dhATX and dhHTX, a pattern also widely observed in *Phormidium* mats in New Zealand rivers [14]. It is unknown whether this is specific to mats from New Zealand, as studies in other countries have rarely used analytical methodology which can detect these congeners (e.g., [11,12]). The highest anatoxin concentration (1889 mg kg^−1^ dw) was detected in Mat B (01:10 h) from the Mataura River, and was greater than two-times higher than previously recorded in a nationwide collation of data from New Zealand rivers (previous maximum = 771 mg kg^−1^ dw; [14]).

A novel observation during this study was the remarkable variability in toxin concentrations within a single mat (i.e., among samples collected within 5–10 cm of each other) over relatively short time periods. For example, anatoxin concentrations in Mat B from the Mataura River at 23:10 and 05:10 were 68-fold higher and 58-fold lower, respectively, than the sample collected at the in-between time of 01:10 (when assessed per dw). The normalization of these data to toxin quota demonstrated this difference was not due to any up- or down-regulation in toxin production, but rather due to the sample collected at 01:10 having an extremely high number of toxic cells. Although we selected defined mats which were separated from those growing on nearby cobbles, it is likely that the mats were initiated from multiple toxic and non-toxic genotypes which grow out of cracks and crevices following the removal or dispersal processes [34]. In the present study we did not enumerate non-toxic *Phormidium* cells and, therefore, cannot assess the relative abundance of toxic versus non-toxic cells, nor did we determine the portion of co-occurring organisms/debris in the samples. If larger samples could have been taken this would enable methods, such as ash-free dry weight, to be used to determine the inorganic content of the mats, and future studies could design *Phormidium* specific primers (e.g., targeting 16S rRNA or RNA polymerase genes), or general eukaryotic PCR assays could be used to determine the amount of co-occurring organisms. Although we cannot rule out the contribution of these factors to the variability in anatoxin concentration, our analysis clearly shows that one of the key contributors to differences in the anatoxin concentrations of mat samples is the abundance of toxic genotypes.

There was considerable variability in anatoxin quotas among samples at each site. This could, in part, be due to an up- or down-regulation in anatoxin production; however, given that no consistent patterns were observed, this is more likely due to differences in the amount of toxin produced by different strains of toxic *Phormidium* cohabitating in the mats. Wood et al. [27] isolated and cultured 30 strains of *Phormidium* from four small sections of benthic mats. Among the toxic strains there were 100-fold differences in the amount of anatoxin produced. Similar strain-to-strain variability in toxin quotas has been shown in planktonic cyanobacteria species [29,35,36,37]. This inter-strain variability makes investigating factors that regulate toxin production in the field challenging and future studies will need to couple the methods used in this study with metatranscriptomic or targeted gene expression approaches. 

Anatoxin quotas were significantly higher in mats from the Mataura River. Both DIN and DRP were also higher in the Mataura River and this may be causing an up-regulation in toxin production. However, this contradicts the observations of Health et al. [26] who, using a single strain and a culture-based study, showed higher anatoxin quotas under low nitrogen and phosphorus regimes. A further possibility is that the sites contain different strains, each with varying capacity to produce anatoxin. In this study we did not observe any strong relationships between the measured physiochemical variables and shifts in anatoxin quotas or gene copy numbers. This may, in part, be because we only measured conditions in the overlying river water, rather than inside the mats, which have been shown to be considerably different [25]. Further targeted field studies or laboratory-based experiments are required to confirm this observation.

## 4. Materials and Methods

### 4.1. Sampling Sites

**Figure 4:**
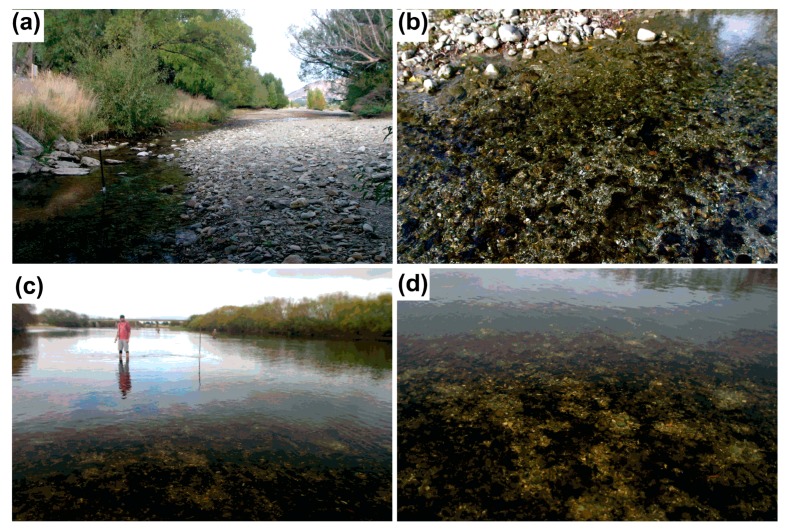
**a**) Sampling site at the Cardrona River; (**b**) *Phormidium* mats in shallow riffle in the Cardrona River (depth ca. 0.15 m); (**c**) sampling site at the Mataura River; and (**d**) *Phormidium* mats on the Mataura River bed (depth ca. 0.5 m).

Field sampling was undertaken at two rivers in the South Island of New Zealand. The Cardrona River (44°41′57′ S, 169°10′38′ E) sampling site was relatively narrow (5–8 m) and largely in riffle habitat with an average depth of 0.4 m (Figure 4a,b). The substrate was small to medium cobbles with approximately 25% of the substrate covered by *Phormidium* mats (Figure 4a,b). Proliferations of *Phormidium* have been reported in this stream during the summer months for the last 3–5 years. The sampling site in the Mataura River (46°23′25′ S, 168°46′35′ E) was approximately 70 m wide and in run habitat (Figure 4c,d). The substrate was small cobbles and approximately 50% of the river bed was covered with *Phormidium* mats (Figure 4c,d). Proliferations of *Phormidium* have been reported in this stream since the early 2000s [38].

### 4.2. Sample Collection

Three mats spaced approximately 10 m apart were selected at each site and coloured markers placed adjacent to them to ensure the correct mat was sampled at each time-point. In the Cardrona River, the selected mats covered a single medium-sized (ca. 20 cm dia.) cobble whereas, in the Mataura River, the selected mats covered 2–3 small (ca. 5 cm dia.) cobbles. In the Cardrona River, samples were collected every 2 h for 24 h, starting at 13:10 h on 10 April 2017. In the Mataura River, mat samples were collected every 2 h (or 3 h at 03:45 and 06:45) for 26 h, starting on 12 April 2017. At each sampling time, a small section (ca. 2 cm diameter) of mat was carefully removed using sterile tweezers, placed in a sterile tube (2 mL), and frozen (−20 °C) for later analysis.

At each sampling time, a water sample (ca. 50 mL) was collected adjacent to the most downstream mat at each site and syringe-filtered (Whatman GF/C, ca. 1.6 µm pore size) and the filtrate frozen (−20 °C) for dissolved nutrient analysis. Temperature was measured at 5 min intervals using a temperature logger (HOBO, Onset, Bourne, MA, USA) positioned on a steel stake approximately 5 m below the most downstream mat. Dissolved oxygen, conductivity and pH were measured adjacent to the temperature logger using a handheld water quality sonde (YSI ProPlus, YSI Inc., Yellow Springs, OH, USA) at each sampling time. The water velocity at each site was measured using a hand held velocity meter (Marsh-McBirney, HACH, Loveland, CO, USA) at the start, middle, and end of the sampling periods.

### 4.3. Laboratory Analysis

The frozen *Phormidium* mat samples were defrosted, transferred to 20 mL glass vials and lyophilized (Gamma 1-16 LSC freeze-drier; Martin Christ Gefriertrocknungsanlagen, Germany). Lyophilized material was ground to a fine powder with a sterile metal spatula and two aliquots were taken. The first aliquot (ca. 10 mg) was weighed (in g to 4 dp; decimal places) into a micro-centrifuge tube and suspended in 1 mL of 0.1% formic acid (made up in Milli-Q water (Merck, Kenilworth, NJ, USA)). These samples were mixed on a vortex (30 s), frozen (−20 °C), and thawed in a sonicator bath (30 min). This freeze-thaw process was repeated two more times before the anatoxin extracts were clarified by centrifugation (12,000× *g*, 5 min) [5]. The clarified extracts were diluted 1/20 in 0.1% formic acid and stored frozen (−20 °C) in glass LC vials until anatoxin analysis.

The second aliquot (ca. 20 mg) was weighed (in g to 4 dp) into the first tube of the PowerMax^®^ Soil DNA Isolation Kit (QIAGEN, Hilden, Germany) and DNA extraction performed following the manufacturer’s protocol. The quantity and quality of the extracted DNA were assessed using a nanophotometer (Implen, München, Germany).

Each anatoxin extract was analysed for ATX, HTX, dhATX and dhHTX by LC-MS/MS, as described in Wood et al. [5]. Briefly, compounds were separated at 40 °C on a Thermo Hypersil Gold aq. column (1.9-µm; 50 × 2.1 mm; Waltham, MA, USA) using a Waters Acquity I-Class ultra-performance LC system (Milford, MA, USA). The mobile phase A (0.1% formic acid in deionized water) and mobile phase B (0.1% formic acid in acetonitrile) were used at a flow of 0.6 mL min^−1^, isocratic for 45 s at 100% A followed by a gradient to 50% B over 3.5 min. The injection volume was 5 µL and 70% acetonitrile was used as the needle wash solvent. The Waters Xevo TQ-S triple quadrapole mass spectrometer was operated in positive-ion mode (100 °C; capillary 0.6 kV; nitrogen desolvation gas 1000 L h^−1^ (450 °C); cone gas 150 L h^−1^). Quantitative analysis was by multiple-reaction monitoring using channels set up for ATX (166.15 > 149.1; retention time (rt) 1.30 min), HTX (180.2 > 163; rt 1.53 min), dhATX (168.1 > 56; rt 1.28 min), and dhHTX (182.1 > 57; rt 1.51 min). Anatoxin concentrations were determined using an external standard curve constructed using dilutions of a certified reference material for ATX (National Research Council, Ottawa, ON, Canada; 0.5–20 ng mL^−1^ in 0.1% formic acid). A relative response factor of 1, using ATX as the calibration reference was used to quantify HTX, dhATX and dhHATX. Data were converted to µg g^−1^ (equivalent to ng mg^−1^) by dividing the LC-MS/MS results (in ng mL^−1^) by the weight of lyophilized starting material (in mg). Anatoxin quotas were calculated by summing all anatoxin variants, and dividing by *anaC* gene copies g^−1^ (see below).

Absolute concentrations of the *anaC* gene were measured in all samples using a BioRad QX200 Droplet Digital PCR system (Hercules, CA, USA) and *Phormidium autumnale* specific primers and a probe (Phor-AnaC-F5 5′-ACTAACCGAATCACTTCCACTT-3′, Phor-AnaC-R5 5′-CTCACCCACCTCACCTTTAG-3’, Phor-AnaC-P5 5′-TTCAGTATTAGCGCAGGCTTTGCC-3′, Laura Kelly, unpublished data). The hydrolysis probe was dual-labelled with a (6-FAM) fluorescent tag (5′ 6-carboxyfluorescein) and a 3′ Black Hole Quencher. Each ddPCR reaction included 450 nM of each primer and probe, 1× BioRad ddPCR Supermix for probes, 1 µL DNA, and sterile water for a total reaction volume of 22 µL. The BioRad QX200 droplet generator partitioned each reaction mixture into nanodroplets by combining 20 µL of the reaction mixture with 70 µL of BioRad droplet oil. After processing, this resulted in a total nanodroplet volume of 40 µL, which was transferred to a PCR plate for amplification using the following cycling protocol: hold at 95 °C for 10 min, 40 cycles of 94 °C for 30 s, 60 °C 1 min, and a final enzyme deactivation step at 98 °C for 10 min. The plate was then analysed on the QX200 instrument. For each ddPCR plate run, at least one negative control (RNA/DNA-free water; UltraPure^TM^, Life Technologies, Carlsbad, CA, USA) and one positive control (genomic DNA extracted from a sample known to contain anatoxin) were included. When inhibition was observed, or samples were too concentrated, these were diluted with RNA/DNA-free water and reanalyzed. The results where then converted to copies g^−1^ using the following formula; number of copies per µL × 22 µL (the initial volume of the PCR reaction) × 100 µL (the volume used to elute the DNA during extraction)/weight of lyophilized starting material (in g).

Water samples were analyzed at Hill Laboratories (Hamilton, New Zealand) with a Lachat Quickchem^®^ flow injection analyzer (FIA+8000 Series, Zellweger Analytics Inc., League City, TX, USA) using [38] 4500 methods for nitrite (NO_2_-N), nitrate (NO_3_-N), ammonium (NH_4_-N) and dissolved reactive phosphorus (DRP) [39]. The limits of quantification were 0.002 mg L^−1^ for NO_2_-N, 0.002 mg L^−1^ for NO_3_-N, 0.01 mg L^−1^ for NH_4_-N, and 0.004 mg L^−1^ for DRP.

### 4.4. Statistical Analysis

Statistical analyses were performed in the software R Studio (Version 3.1.1 [40]). Student *t*-tests were used to compare means of anatoxin concentrations, anatoxin quotas, and DIN concentrations between the Cardrona and Mataura rivers. Normality was checked through inspection of Quantile-Quantile plots and conducting a Shapiro-Wilk test. As normality was not met for anatoxin concentrations and anatoxin quota data, a log transformation was undertaken. Linear regression was used to explore potential relationships between anatoxin quotas and *anaC* copy numbers and measured physiochemical parameters.

## 5. Conclusions

The results of this study confirm that the abundance of anatoxin-producing strains within a benthic mat are one of the main contributors to the dramatic spatial and temporal differences in anatoxin concentrations of *Phormidium*-dominated mats observed in this study, and during previous, studies. The data from the present study showed that high spatial variability of anatoxin concentrations in *Phormidium* mats occurred at a finer scale than previously demonstrated (cm rather than m [27,41]). When undertaking risk assessments these data reinforce suggestions from previous studies [22], that multiple mat samples must be collected at a sampling site to provide an accurate assessment of anatoxin concentrations at that location. When coupled with ‘omics’ techniques, the sample preparation methods used during the present study will provide further insights into the factors regulating anatoxin production in benthic *Phormidium* mats.

## Supplementary Materials

The following are available online at www.mdpi.com/1660-3397/15/10/307/s1. Figure S1. Temperature measured every 5 min over the sampling period in the Cardrona River and Mataura River; Table S1. Physiochemical parameters measured over the sampling period in the Cardrona River and the Mataura River.
