# Supplementary material for: The Abundance of Toxic Genotypes Is a Key Contributor to Anatoxin Variability in Phormidium-Dominated Benthic Mats
Source: Mar Drugs. 2017 Oct 11;15(10):307. doi: 10.3390/md15100307 (PMC5666415; doi:10.3390/md15100307)
Supplement: Supplementary file 1 [file marinedrugs-15-00307-s001.pdf]

Supplementary Information Figure 1. Temperature measured every 5 min over the sampling period in (**a**) the Cardrona River (10–11 April 2017 and (**b**) the Mataura River (12–13 April 2017.

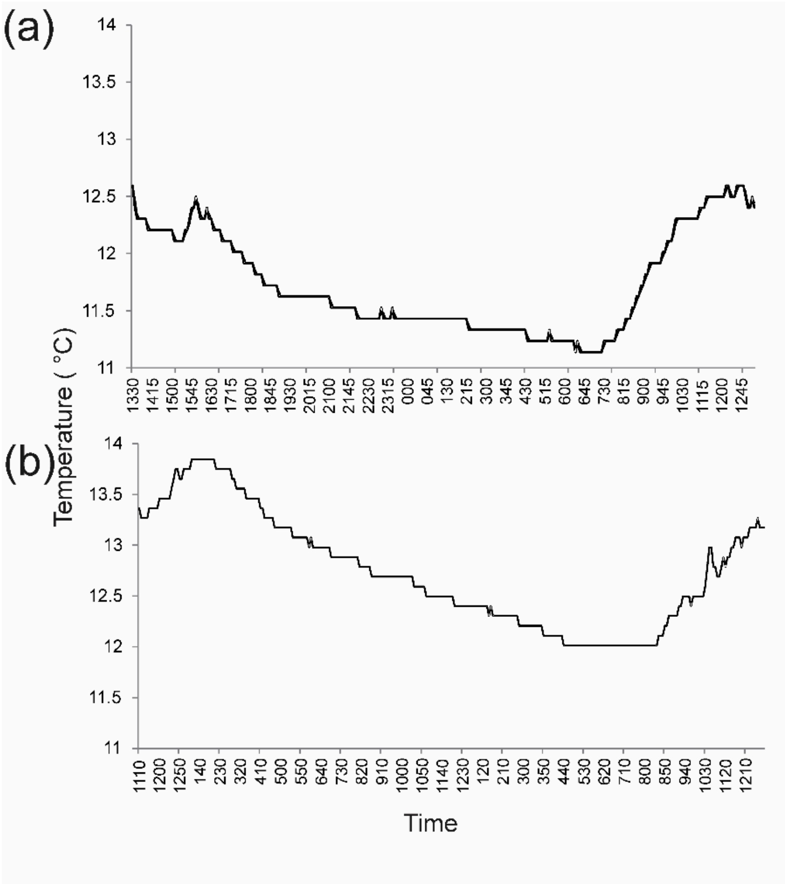

Supplementary Information Table 1. Physiochemical parameters measured over the sampling period in the Cardrona River (10–11 April 2017), and Mataura River (12–13 April 2017). DIN = dissolved inorganic nitrogen; DRP = dissolved reactive phosphorus.

Cardrona River							Mataura River						
Date	Time	Diss. O^2 (%)	Cond. (μS cm^{-1})	pH	DIN (mg L^{-1})	DRP mg L^{-1})	Date	Time	Diss. O^2 (%)	Cond. (μS cm^{-1})	pH	DIN (mg L^{-1})	DRP (mg L^{-1})
10 Apr 2017	1310		108.70	7.56	0.136	<0.004	11 Apr 2017	1045	102.30	113.8	7.26	0.504	0.006
10 Apr 2017	1510	73.6	108.30	7.18	0.215	<0.004	11 Apr 2017	1245	114.80	114	7.98	0.684	0.006
10 Apr 2017	1710	73.2	108.30	7.01	0.401	<0.004	11 Apr 2017	1445	119.80	114.4	8.28	0.100	0.005
10 Apr 2017	1910	73.2	108.40	7.05	0.295	<0.004	11 Apr 2017	1645	110.20	115.8	7.79	0.782	0.01
10 Apr 2017	2110	73.9	108.70	7.02	0.355	<0.004	11 Apr 2017	1845	100.90	116.6	7.47	0.711	0.005
10 Apr 2017	2310	72.1	108.40	7.1	0.435	<0.004	11 Apr 2017	2045	95.40	118	7.41	0.564	0.009
11 Apr 2017	0110	71.3	108.50	7.2	0.385	0.004	11 Apr 2017	2245	93.80	119.3	7.31	0.714	0.008
11 Apr 2017	0310	71.4	107.40	7.15	0.405	<0.004	11 Apr 2017	1245	91.60	119.6	7.31	0.572	0.006
11 Apr 2017	0510	73.1	108.60	7.15	0.464	<0.004	12 Apr 2017	0345	90.50	120.9	7.2	0.639	0.007
11 Apr 2017	0710	71.1	107.00	7.25	0.176	<0.004	12 Apr 2017	0645	89.10	123.9	7.29	1.114	0.011
11 Apr 2017	0910	76.4	106.00	7.24	0.183	<0.004	12 Apr 2017	0845	99.00	123	7.26	0.306	0.006
11 Apr 2017	1110	79.2	108.40	7.16	0.403	<0.004	12 Apr 2017	1045	112.40	126.2	7.57	0.355	0.006
11 Apr 2017	1310		108.60	7.01	0.355	<0.004	12 Apr 2017	1245	121.60	132	7.51	0.458	0.011
